# Effects of systemic inflammation on relapse in early breast cancer

**DOI:** 10.1038/s41523-020-00212-6

**Published:** 2021-01-22

**Authors:** Nicholas P. McAndrew, Lisa Bottalico, Clementina Mesaros, Ian A. Blair, Patricia Y. Tsao, Jennifer M. Rosado, Tapan Ganguly, Sarah J. Song, Phyllis A. Gimotty, Jun J. Mao, Angela DeMichele

**Affiliations:** 1 Perelman School of Medicine at the University of Pennsylvania, Philadelphia, PA 19104 USA; 2 Division of Hematology/Oncology, Department of Medicine, University of Pennsylvania, Philadelphia, PA 19104 USA; 3 Abramson Cancer Center, University of Pennsylvania, Philadelphia, PA 19104 USA; 4 Department of Systems Pharmacology and Translational Therapeutics, University of Pennsylvania, Philadelphia, PA 19104 USA; 5 Department of Biostatistics, Epidemiology and Informatics, University of Pennsylvania, Philadelphia, PA 19104 USA; 6 Department of Medicine, Memorial Sloan-Kettering Cancer Center, New York, NY 10065 USA

**Keywords:** Prognostic markers, Breast cancer

## Abstract

Chronic inflammation has been a proposed mechanism of resistance to aromatase inhibitors in breast cancer. Stratifying by HER2 status, a matched case-control study from the Wellness After Breast Cancer-II cohort was performed to assess whether or not elevated serum inflammatory biomarkers (C-Reactive protein [CRP], interleukin-6 [IL-6], and serum amyloid A [SAA]) and/or the presence of a high-risk IL-6 promoter genotype were associated with recurrence of hormone receptor positive (HR+) early breast cancer. Estrogen levels were also measured and correlated with biomarkers and disease outcomes. CRP and SAA were significantly associated with an increased risk of recurrence in the HR+/HER2− group, but not the HR+/HER2+ group. Mean serum estrogen levels were non-significantly elevated in patients who relapsed vs. non-relapsed patients. Surprisingly, high-risk IL-6 promoter polymorphisms were strongly associated with HER2+ breast cancer relapse, which has potential therapeutic implications, as elevated intracellular IL-6 has been associated with trastuzumab resistance in pre-clinical models.

## Introduction

Estrogen receptor positive (ER+) breast cancer represents approximately 60–80% of all breast cancer diagnoses^1^. Aromatase inhibitors (AIs) have revolutionized treatment for this disease by blocking the peripheral conversion of androgens to estrogen, primarily in adipose tissue^2^ thereby reducing recurrence and improving survival in the adjuvant setting. These benefits extend to both post-menopausal and high-risk premenopausal women (when combined with ovarian suppression). Nevertheless, up to 30% of women will recur during their lifetimes with a persistent and cumulative risk of relapse^2^.

The mechanisms underlying failure of AIs in this setting are poorly understood^3^, but appear to be multifactorial, and there is prior evidence that inflammation may play a significant role^4^. While there are multiple inflammatory pathways and molecules that have been implicated in ER+ breast cancer recurrence^5–7^, the inflammatory cytokine interleukin-6 (IL-6) and its associated pathway has been shown to be associated with poor outcomes in ER+ breast cancer^8^. Functional polymorphisms in the IL-6 promoter that result in increased transcription of IL-6 are significantly associated with worse prognosis and decreased disease free survival (DFS) in high-risk breast cancer patients^9^. Increased production of IL-6 has both cellular and microenvironmental effects in the breast, including upregulated activity of aromatase and other enzymes involved in post-menopausal estrogen biosynthesis^8^. In addition, the soluble IL-6 receptor is expressed at higher levels in many ER+ breast cancers as compared to the triple negative subtype^4^, enabling IL-6 to impact intracellular signaling through the JAK/STAT pathway.

Two downstream products of IL-6 activation, C-reactive protein (CRP) and serum amyloid A (SAA), are mainly produced by the liver, and circulate systemically^4^. While serum CRP and SAA levels generally rise with increasing serum IL-6 levels, they can be differentially elevated in different disease states^10^, suggesting somewhat distinct production pathways. High levels of serum CRP and SAA have been associated with increased risk of relapse and decreased survival in large, prospective cohort studies that include all molecular subtypes of breast cancer^11,12^.

Given the links between IL-6, its downstream effectors CRP and SAA, and aromatase activity, we hypothesized that patients with high circulating levels of these inflammatory cytokines, as well as patients with high-risk IL-6 promoter genotypes, may be more likely to recur during or after treatment with an aromatase inhibitor compared to those without elevated inflammatory markers. Furthermore, we hypothesized that increased serum estrogen metabolites would be associated with both increased inflammatory markers, as well as increased risk of recurrence. We examined these questions in a cohort of women with ER+ breast cancer who received an AI in the adjuvant setting and for whom long-term disease outcomes were available.

## Results

### HER2− cohort

Patients for this nested case-control study were identified from among a total of 1287 patients enrolled to the parent cohort, the Wellness After Breast Cancer-II (WABC-II) cohort study. WABC-II was designed, as previously described^13,14^, as a prospective non-intervention cohort study with the specific aims of investigating the risk factors and underlying mechanisms of two categories of outcomes: symptom distress including AI-induced arthralgia, and disease outcomes including failure of AI therapy. To examine early treatment failure specifically, we identified a total of 197 unique cases consisting of those patients with stage I–III ER+ breast cancer who relapsed on or after adjuvant AI and matched controls who remained free of recurrence during or after AI therapy for this study. Of these, 123 tumors were HER2−, while 74 tumors were HER2+. Given the likelihood that risk factors for recurrence differ between HER2+ and HER2− ER+ patients, we analyzed these groups separately.

There were 5 missing samples from among the HER2− subjects (1 case and 4 controls), resulting in 118 analyzable cases in the HR+/HER2− group (38 cases and 80 matched controls) for this analysis. Demographics are reported in Table 1. Amongst the HER2− cases and controls, there was a significant difference in nodal status (29% vs. 10%, respectively, *p* = 0.009), and while cases were generally of higher grade, this difference was of borderline significance (26% vs. 13%, respectively, *p* = 0.062).

**Table 1:** Clinical characteristics of HER2− and HER2+ patients.

Group	HER2−			HER2+		
Covariate	Controls (*n* = 80)	Cases (*n* = 38)	*p*	Controls (*n* = 56)	Cases (*n* = 11)	*p*
Age (median)	55 years	52 years	0.330	57.5 years	60 years	0.972
BMI (≥30)	26 (35%)	11 (31%)	0.665	14 (25%)	3 (27%)	0.842
Nodes (>4)^a^	8 (10%)	11 (29%)	0.009	9 (16%)	2 (18%)	0.863
Race						
White	61 (76%)	31 (81%)	0.514	42 (75%)	7 (64%)	
Black	11 (14%)	6 (16%)		9 (16%)	3 (27%)	
Other/unknown	8 (10%)	1 (3%)		5 (9%)	1 (9%)	0.437
High-grade	10 (13%)	10 (26%)	0.062	24 (43%)	6 (46%)	0.476

*BMI* body mass index

^a^*p* < 0.05 in HER2− group

#### Inflammatory biomarkers

Serum inflammatory biomarker results are shown in Table 2. The overall mean, median, and range was 11.33, 4.83, 0.30–88.49 for CRP (mg/L), 13.76, 7.91, 0.58–119.37 for SAA (mg/L), and 3.16, 2.56, 0.02–18.07 for IL-6 (pg/mL). CRP and SAA levels were moderately correlated with each other (correlation coefficient 0.404), while there was little to no correlation between IL-6 and CRP (0.135) or SAA (0.040) levels. Cases had significantly higher median serum levels of CRP relative to controls (9.54 vs. 3.25 mg/L, respectively, *p* = 0.004) and SAA (11.03 vs. 6.81 mg/L, respectively, *p* = 0.009), while serum IL-6 concentrations did not differ (2.66 pg/mL in cases vs. 2.52 pg/mL in controls, *p* = 0.7911). In addition, cases had a higher proportion of subjects with CRP values >ULN (>7.5 mg/L) relative to controls (55% vs. 29%, respectively, *p* = 0.005). The proportion of subjects with IL-6 levels >ULN (≥5 pg/mL) did not differ between cases and controls (11% vs. 16%, respectively, *p* = 0.408). Univariate conditional logistic regression models (Table 3) showed that having a serum CRP or SAA level ≥ the median value was significantly associated with breast cancer relapse, with CRP OR 2.4 (95% confidence interval (CI) 1.16–5.00, *p* = 0.019) and SAA OR 3.38 (95% CI 1.57–7.25, *p* = 0.002). IL-6 levels greater than or equal to the median value were not associated with an increased risk of relapsed breast cancer (OR 1.38, 95% CI 0.68–2.82, *p* = 0.367). Similarly, subjects with CRP levels >ULN were more likely to have relapsed breast cancer (OR 2.66, 95% CI 1.34–5.28, *p* = 0.005), while subjects with IL-6 levels >ULN had no increased likelihood of relapsed breast cancer (OR 0.62, 95% CI 0.20–1.90, *p* = 0.402).

**Table 2:** Biomarker concentrations by case status and HER2 status.

Group	HER2−			HER2+		
Biomarker	Controls (*n* = 80)	Cases (*n* = 38)	*p*	Controls (*n* = 56)	Cases (*n* = 11)	*p*
Median CRP mg/L (r)	3.25 (0.30–61.65)	9.54 (0.60–88.49)	0.0042	3.70 (0.68–222.09)	7.30 (0.88–49.50)	0.5423
Median IL-6 pg/mL (r)	2.52 (0.38–18.07)	2.66 (0.02–7.59)	0.7911	2.59 (0.32–10.72)	2.68 (1.47–15.81)	0.3617
Median SAA mg/L (r)	6.88 (0.81–119.37)	11.03 (0.57–116.18)	0.0089	10.94 (1.48–384.58)	10.74 (2.08–30.48)	0.8337
CRP > 7.5 mg/L (%)	29%	55%	0.005	25%	36%	0.437
IL-6 > 5 pg/mL (%)	16%	11%	0.408	6%	9%	0.694

*r* range

**Table 3:** Univariate logistic* regression of inflammatory biomarkers.

Group	HER2−		HER2+	
Biomarker	OR (95% CI)	*p*	OR (95% CI)	*p*
Log CRP	1.68 (1.25–2.26)	0.001	1.10 (0.69–1.77)	0.683
Log IL6	0.98 (0.64–1.52)	0.940	1.99 (0.69–5.73)	0.201
Log SAA	1.79 (1.18–2.72)	0.007	0.85 (0.43–1.70)	0.655
CRP ≥ median	2.41 (1.16–5.00)	0.019	1.88 (0.49–7.15)	0.354
IL-6 ≥ median	1.37 (0.68–2.82)	0.367	1.25 (0.34–4.61)	0.740
SAA ≥ median	3.38 (1.57–7.25)	0.002	0.77 (0.21–2.84)	0.699
CRP > ULN	2.66 (1.34–5.28)	0.005	1.71 (0.43–6.74)	0.440
IL-6 > ULN	0.62 (0.20–1.90)	0.402	1.60 (0.15–17.00)	0.697
CRP > 10.3 mg/L	4.22 (2.00–8.90)	<0.001	NP	NP
SAA > 7.3 mg/L	4.22 (1.77–10.09)	0.008	NP	NP

*ULN* upper limit of normal

NP = Not performed due to lack of association in preliminary analysis

Median CRP (mg/L) in the HER2− group = 4.83 and 3.78 in the HER2+ group

Median IL-6 (pg/mL) in the HER2− group = 2.56 and 2.60 in the HER2+ group

Median SAA (mg/L) in the HER2− group = 7.91 and 10.92 in the HER2+ group

*Conditional logistic regression for HER2− group

**Fig. 1:**
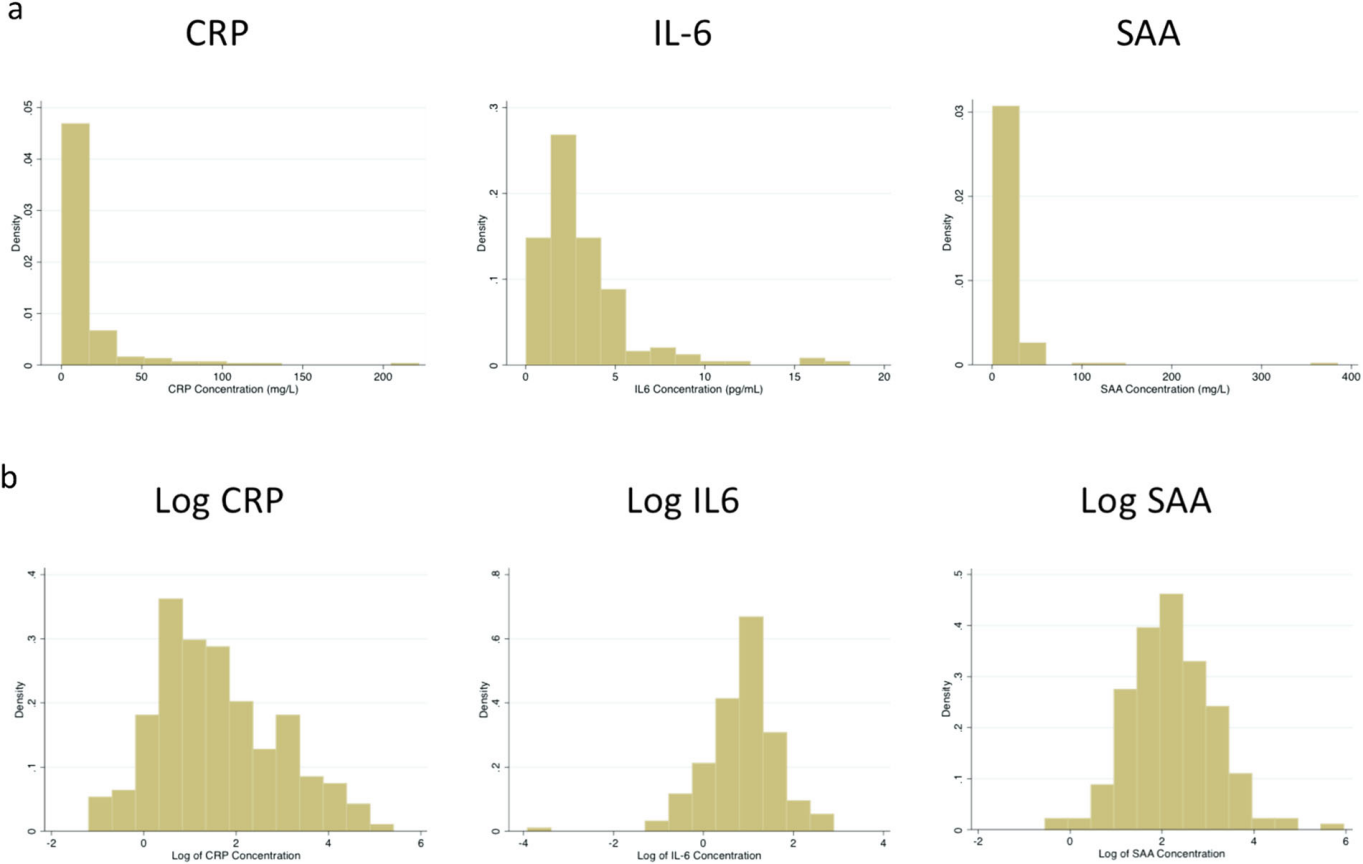
Biomarker histograms. **a** Serum biomarker concentrations; **b** Natural log transformed serum biomarker concentrations.

**Fig. 2:**
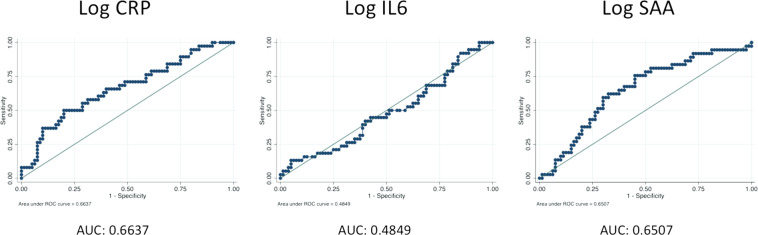
Receiver operating characteristic curves for each inflammatory biomarker (HER2− subjects).

**Fig. 3:**
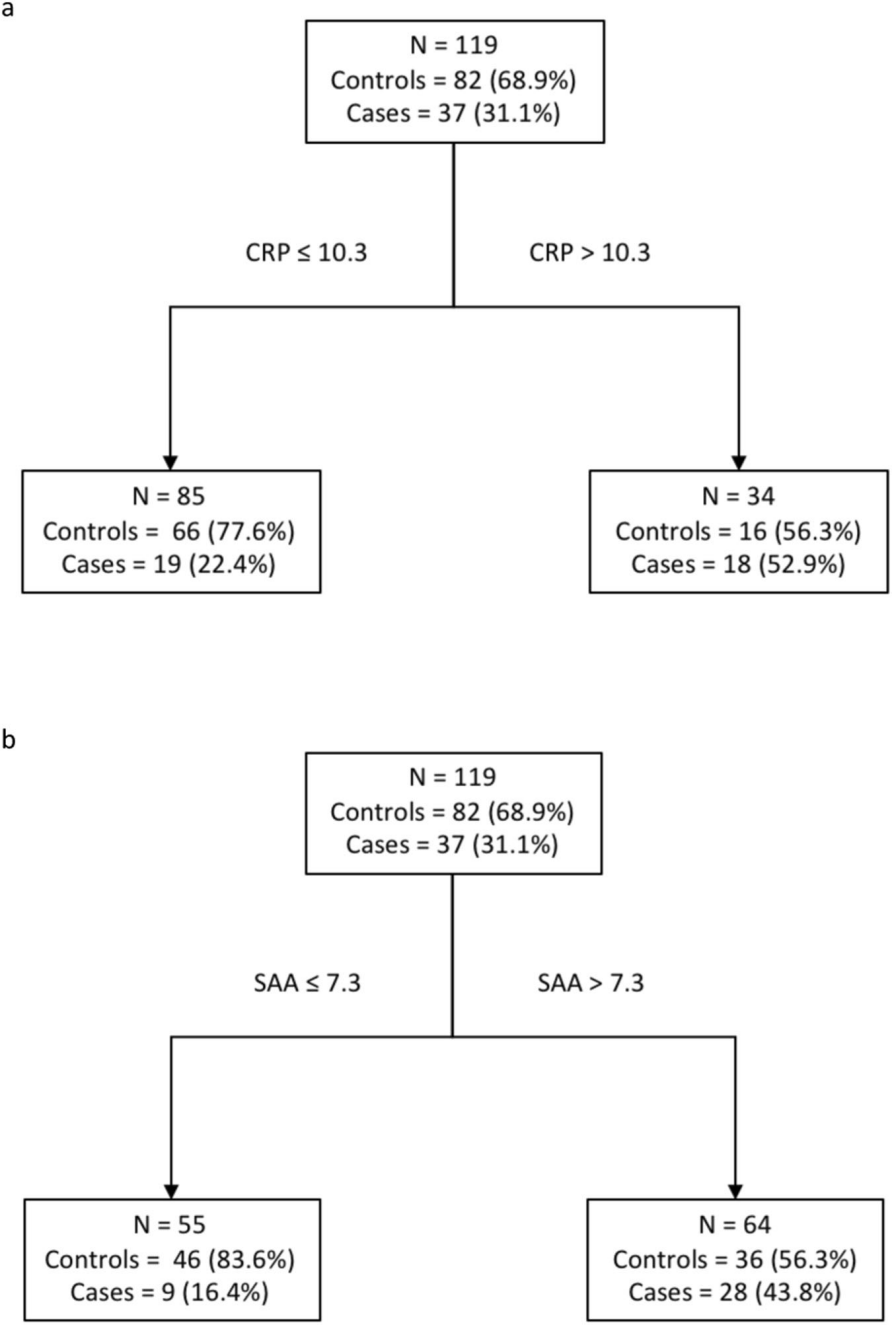
Classification trees (HER2− subjects). **a** CRP; **b** SAA.

Continuous measures of inflammatory biomarkers in non-transformed and natural log transformed distributions are shown in Figs 1a, b, and Table 3 shows the univariate conditional logistic regression results. Increasing CRP and SAA levels were associated with a significantly increased risk of relapse. For CRP, the OR was 1.68, (95% CI 1.25–2.26, *p* = 0.001); for SAA, the OR was 1.79 (95% CI 1.18–2.72, *p* = 0.007). Increasing IL-6 levels were not associated with increased risk of breast cancer relapse (OR 0.98, 95% CI 0.64–1.52, *p* = 0.940). ROC curves using these continuous measures, as shown in Fig. 2, show that both CRP and SAA have moderate predictive ability to discriminate cases from controls (area under the curve 0.6637 and 0.6507, respectively). Classification trees generated separately for CRP and SAA (Fig. 3a, b, respectively) to determine the value of each inflammatory protein above which the proportion of cases was maximized demonstrated that serum CRP level greater than 10.3 mg/L and a serum SAA level >7.3 mg/L were determined to be the cutpoints that best classified cases from controls. The univariate conditional logistic regression for each of these cutpoints (Table 3) show that serum CRP level >10.3 mg/L was associated with a significantly increased risk of relapse (OR 4.22, 95% CI 2.00–8.91, *p* < 0.001), with similar results for serum SAA >7.3 mg/L (OR 4.22, 95% CI 1.77–10.09, *p* = 0.008).

**Fig. 4:**
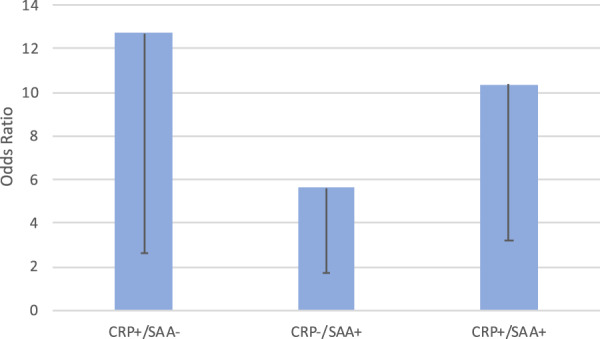
Multivariable conditional logistic regression of CRP and SAA and risk of relapsed breast cancer (additive scale, HER2− subjects). Description: Error bars represent lower 95% confidence interval.

To test for statistical (multiplicative) interaction between CRP and SAA, the individual dichotomous variables of CRP and SAA, as well as the cross-product of CRP*SAA were combined in a multivariate conditional logistic regression model, with ORs (and 95% CIs) of 12.70 (2.64–61.10, *p* = 0.002) for CRP, 5.62 (1.74–18.21, *p* = 0.004) for SAA, and 0.15 (0.03–0.84, *p* = 0.031) for CRP*SAA. A test for synergy on the additive scale did not suggest a synergistic relationship between CRP and SAA in predicting increased risk of breast cancer relapse. Variables considered in the biologic (additive) analysis are represented as CRP+/SAA+, CRP+/SAA−, and CRP−/SAA+(with CRP−/SAA− as the reference group). Results are shown in Fig. 4. The OR for CRP+/SAA+ was 10.37 (95% CI 3.21–33.56, *p* < 0.001. The RERI was −6.95 (95% CI −25.43 to 11.53).

To determine whether the clinical covariates listed in Table 1 were potential confounders or effect modifiers, bivariate conditional logistic regressions for relapsed breast cancer, as well as levels of CRP/CAA (dichotomized at the classification tree cutpoints described above) as the dependent variable was performed with each covariate as independent variables (Tables 4 and 5, respectively). Of note, 26% of subjects (40/118 in the HER2− group and 7/67 in the HER2+ group) did not have grade specified in the pathology report. The covariates significantly associated with increased risk of relapsed breast cancer were >4 positive nodes (OR 2.35, 95% CI 1.07–5.18, *p* = 0.034) and high-grade histology (OR 2.95, 95% CI 1.17–7.41, *p* = 0.022). BMI ≥ 30 kg/m^2^ was not associated with increased risk of relapsed breast cancer, but was the only covariate significantly associated with any of the elevated serum levels of CRP (OR 5.07, 95% CI 2.09–12.33, *p* < 0.001) and SAA (OR 2.47, 95% CI 1.06–5.78, *p* = 0.036). A significantly higher proportion of patients with a BMI ≥ 30 kg/m^2^, compared to those with BMIs <30 kg/m^2^, had elevated levels of CRP (50% vs. 24%, *p* = 0.005) and serum SAA (42% vs. 23%, *p* = 0.034). Based on these analyses, nodal status and high-grade pathology were included as candidate covariates in the final multivariate conditional logistic regression model.

**Table 4:** Associations of covariates with relapsed breast cancer, by HER2 cohort.

Group	HER2−		HER2+	
Covariate	OR (95% CI)	*p*	OR (95% CI)	*p*
BMI ≥ 30	1.12 (0.52–2.40)	0.781	1.39 (0.36–5.42)	0.633
Nodes (>4)	2.35 (1.07–5.18)	0.034	1.05 (0.20–5.55)	0.954
Age (≥median)	0.98 (0.46–2.09)	0.948	1.21 (0.36–4.00)	0.760
Race (White vs. Non-White)	1.18 (0.48–2.93)	0.710	0.35 (0.10–1.20)	0.096
High-grade	2.95 (1.17–7.41)	0.022	1.08 (0.32–3.59)	0.901

*BMI* body mass index

**Table 5:** Associations of covariates with elevated inflammatory biomarkers (HER2− group only).

Biomarker	CRP > 10.3 mg/L		SAA > 7.3 mg/L	
Covariate	OR (95% CI)	*p*	OR (95% CI)	*p*
BMI ≥ 30	5.07 (2.09–12.33)	<0.001	2.47 (1.06–5.78)	0.036
Nodes (>4)	0.82 (0.27–2.49)	0.728	1.52 (0.55–4.18)	0.420
Age (≥median)	0.50 (0.22–1.12)	0.093	0.69 (0.33–1.42)	0.312
Race (White vs. Non-White)	0.48 (0.19–1.19)	0.114	1.05 (0.44–2.51)	0.921
High-grade	1.35 (0.49–3.73)	0.567	1.01 (0.39–2.67)	0.976

*BMI* body mass index

The significant candidate biomarkers CRP and SAA, the cross-product interaction term of CRP*SAA, and the covariates associated with increased risk of breast cancer relapse were added to a multivariate conditional logistic regression model using both forward and backward variable selection methods. In the final model, nodal status was not significantly associated with relapsed breast cancer after adjusting for the other variables (Table 6). High levels of CRP and SAA, the multiplicative interaction, CRP*SAA, and high-grade pathology were all significantly associated with increased risk of relapsed breast cancer.

**Table 6:** Final multivariable conditional logistic regression model (HER2− subjects).

Variable	OR (95% CI)	*p*
High CRP (>10.3 mg/L)	17.17 (3.17–92.87)	0.001
High SAA (>7.3 mg/L)	8.26 (2.30–29.60)	0.001
CRP*SAA	0.09 (0.01–0.62)	0.014
High-grade pathology	5.86 (1.74–19.76)	0.004

In order to assess for any potential selection bias that may have occurred as a result of extremes of follow up time, a sensitivity analysis was performed for the HER2− group excluding patients with the 5% least amount of follow up time (<6 years) and the 5% greatest amount of follow up time (>18 years). The results of univariate conditional logistic regression remained similar for elevated CRP (OR 4.65, 95% CI 1.69–121.78, *p* = 0.003), and were more pronounced for elevated SAA (OR 20.85, 95% CI 2.70–161.11, *p* = 0.004).

To achieve the 1:5 case/control matching ratio for every case, controls were replaced back into the selection pool, with controls being used a median of 3 times (range 1–15). Because this may bias the results in favor of the inflammatory profile of controls used multiple times, a sensitivity analysis was performed using simple logistic regression in the HER2− group without the matching criteria so that each control was only used once. The results were similar for elevated CRP (OR 4.00, 95% CI 1.73–9.26, *p* = 0.001) and SAA >7.3 mg/L (OR 3.80, 95% CI 1.59–9.08, *p* = 0.003), and overall similar to the results from the univariate conditional logistic regression analyses.

#### Role of IL-6 promoter genotype

The proportion of HER2− subjects with the high-risk IL-6 promoter genotype profile was slightly higher among cases (58% of cases vs. 51% of controls), though this difference was not statistically significant (Chi squared *p* = 0.499). However, the presence of a high-risk IL-6 promoter genotype was significantly associated with increased risk of relapsed breast cancer in a univariate conditional logistic regression model (OR 2.35, 95% CI 1.16–4.77, *p* = 0.018). Of the clinical covariates significantly associated with relapsed breast cancer (nodal status and high-grade), neither were associated with high-risk genotype profiles (nodal status: OR 1.83, 95% CI 0.87–3.82, *p* = 0.109; high-grade: OR 0.92, 95% CI 0.40–2.08, *p* = 0.835). Using the cutoff values for CRP and SAA generated by the classification tree analysis, patients with high-risk IL-6 promoter genotypes were significantly more likely to have elevated serum SAA (OR 1.97, 95% CI 1.10–3.53, *p* = 0.022), while likelihood of elevated CRP was of borderline significance (OR 1.85, 95% 0.98–3.50, *p* = 0.059) in a univariate conditional logistic regression.

#### Estrogen Metabolites and Aromatase Inhibitor Detection

Detectable levels of serum estrogens were similar between cases and controls, both for E1 (50% of cases and 47% of controls) and E2 (19% of cases and 21% of controls). Among patients receiving standard suppressive doses of an AI, a greater number of subjects with elevated CRP levels (>10.3 mg/L) had detectable levels of serum E1 (57% vs. 45%, respectively) and E2 (26% vs. 17%, respectively) compared to those with lower CRP levels, but these differences did not reach statistical significance (*p* = 0.212 for difference in E1, *p* = 0.268 for difference in E2). High serum SAA level (>7.3 mg/L) was not associated with detectable serum E1 (52% vs. 46%, respectively, *p* = 0.530) or E2 (19% vs. 21%, respectively, *p* = 0.774). Results were similarly negative when stratifying by high-risk IL-6 promoter genotype compared to a non-high-risk genotype profile for both E1 (51% vs. 49%, respectively, *p* = 0.563) and E2 (21% vs. 18%, respectively, *p* = 0.737).

**Fig. 5:**
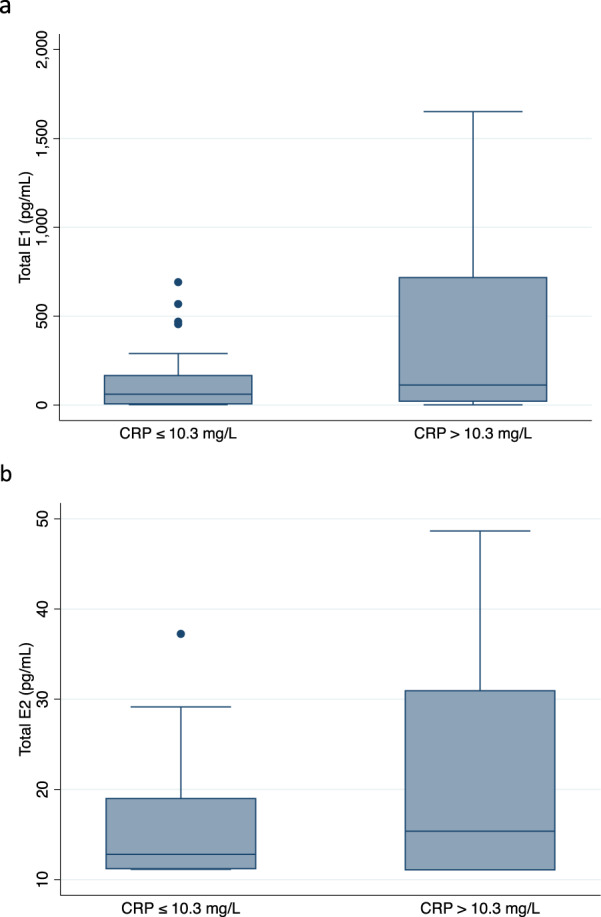
Box graphs of serum estrogen levels by CRP status (HER2− subjects). **a** Detectable E1 levels stratified by high CRP status; **b** Detectable E2 levels stratified by high CRP status. Description: Whisker endpoints represent the range of results (minimum-maximum), box limits represent the interquartile range (between the upper and lower quartiles), center line represents the median.

The mean values were 78.3 pg/mL for E1 and 3.7 pg/mL for E2. Cases relative to controls had higher median values of E1 (104.3 vs. 66.2 pg/mL) and E2 (4.5 vs. 3.3 pg/mL), but these differences were not statistically significant (Wilcoxon rank-sum *p* = 0.638 for difference in E1 and p = 0.866 for difference in E2). Differences in detectable estrogen levels stratified by CRP level are shown in Fig. 5. Subjects with high serum CRP relative to low serum CRP also had higher but not significantly different mean values for E1 (137.5 vs. 53.9 pg/mL, *p* = 0.182) and E2 (5.7 vs. 2.9 pg/mL, *p* = 0.338). Subjects with high-risk compared to non-high-risk IL-6 promoter genotype profiles had non-significantly lower mean values of E1 (73.1 vs. 84.0 pg/mL, *p* = 0.783), with no significant difference in mean E2 (3.2 vs. 4.2, *p* = 0.965).

To evaluate for adherence to therapy, available serum samples (4/118, or 3.3% samples missing) were analyzed for detection of any AI (anastrozole, letrozole, or exemestane). 72.8% of patients had detectable levels of an AI, while 27.2% of patients had no detectable level of an AI. Detection of an AI did not significantly reduce the risk of relapse in a univariate conditional logistic regression (OR 0.75, 95% CI 0.33–1.68, *p* = 0.480).

### HER2+ cohort

There were 7 missing samples from the HR+/HER2+ subjects (1 case and 6 controls), resulting in a total of 67 subjects (11 cases and 56 non-matched controls) in this analysis. Demographics are reported in Table 1. There were no significant differences between the HR+/HER2+ cases and controls with respect to these baseline covariates. None of the baseline covariates were associated with an increased risk of relapse of breast cancer in univariate logistic regression analyses (Table 4).

#### Inflammatory biomarkers

Among patients with HER2+ breast cancer, CRP and SAA levels exhibited a moderate degree of correlation (0.423), while IL-6 levels exhibited little to no correlation with CRP (0.285) or SAA (−0.084). In the HR+/HER2+ group, there were no significant differences between cases and controls in median serum concentrations of CRP (7.30 vs. 3.70 mg/L, *p* = 0.5423), IL-6 (2.68 and 2.59 pg/mL, *p* = 0.3617), or SAA (10.74 vs. 10.94 mg/L, *p* = 0.8337). For the HER2+ group, none of the three candidate biomarkers were associated with relapsed breast cancer in a univariate logistic regression of log transformed serum concentrations of CRP (OR 1.10, 95% CI 0.69–1.77, *p* = 0.683), SAA (OR 0.85, 95% CI 0.43–1.70, *p* = 0.655), or IL-6 (OR 1.99, 95% CI 0.69–5.73, *p* = 0.201). Therefore, no further analyses of the inflammatory biomarkers were considered in the HER2+ group.

#### IL-6 promoter genotype

HER2+ cases and controls differed significantly by the proportion of subjects with a high-risk IL-6 promoter genotype (82% vs. 45%, respectively, *p* = 0.024). In a univariate logistic regression analysis, a high-risk IL-6 promoter genotype profile was significantly and strongly associated with increased risk of relapsed breast cancer (OR 7.19, 95% CI 1.47–35.30, *p* = 0.015). None of the clinical covariates were associated with an increased risk of HER2+ breast cancer relapse in a multivariate logistic regression analysis, either with or without the inclusion of the genotype profile into the model. Hence, the univariate regression of the genotype profile was considered the final regression model.

#### Estrogen metabolites and aromatase inhibitor detection

Of the 66 HR+/HER2+ cases and controls with serum available for estrogen metabolite analysis, cases had a significantly higher proportion of subjects with detectable E1 (7/11, or 63%) as compared to controls (16/55, or 29%, Chi squared *p* = 0.028). Cases also had a significantly higher proportion of subjects with detectable E2 (5/11, or 44%) as compared to controls (8/55, or 15%, Chi squared *p* = 0.019). Mean values were 32.3 pg/mL for E1 and 1.7 pg/mL for E2. Cases relative to controls had non-significantly higher mean levels of E1 (57.4 vs. 28.5 pg/mL, *p* = 0.126) and E2 (3.8 vs. 1.4 pg/mL, *p* = 0.212). Other results of estrogen analyses stratified by CRP and IL-6 promoter polymorphism status in HER2+ subjects are shown in Supplementary Table 1 (supplemental material). HER2+ subjects with a high-risk promoter polymorphism relative to those with a non-high-risk polymorphism had a significantly higher proportion of detectable E2 levels (29% vs. 9%, *p* = 0.041). Otherwise, there were no significant differences in proportion of detectable or mean levels of E1 or E2 when stratifying by median CRP level or high-risk IL-6 promoter genotype profile.

After analyzing available serum samples (14/74, or 18.9% samples missing) for detection of any AI (anastrozole, letrozole, or exemestane), 73.3% of patients had detectable levels of an AI, while 26.7% of patients had no detectable level of an AI. Detection of an AI did not significantly reduce the risk of relapse in a univariate logistic regression (OR 0.30, 95% CI 0.07–1.38, *p* = 0.123).

## Discussion

In summary, these results demonstrate that levels of serum inflammatory proteins CRP and SAA are independently associated with an increased risk of breast cancer relapse in HR+/HER2− breast cancer. A similar association was not seen in HR+/HER2+ tumors. CRP and SAA are not synergistic predictors; rather, the risk of recurrence with elevated CRP alone is similar to that of CRP and SAA. High-grade pathology appears to attenuate this relationship. In addition, carriers of the high-risk IL-6 promoter genotype were significantly more likely to relapse in both HER2− and HER2+ disease, but the magnitude of the association was larger in the ER+/HER2+ subtype. While serum estrogen levels are higher in patients with elevated inflammatory biomarkers in the HER2− subjects, this difference did not reach statistical significance. Non-detection of serum AI levels was not significantly associated with increased risk of relapse in the HER2− or HER2+ groups.

The mechanisms underlying the resistance pathways to adjuvant aromatase inhibitors in HR+ breast cancer remain unclear, and are likely to be multifactorial, involving a combination of both tumor and host factors. AI resistance may be influenced by factors as singular as somatic mutations in malignant cells, to complex interactions with growth factors and inflammatory cytokines produced by the surrounding tumor microenvironment^15^. Identifying therapeutic pathways beyond estrogen deprivation or estrogen receptor blockade that may further reduce an individual’s risk of breast cancer relapse remains an unmet clinical need.

Our findings corroborate and expand upon other reports of the role of host inflammatory response in the risk of breast cancer recurrence. Previous studies to report on the association between serum CRP and SAA and breast cancer relapse/survival did not stratify by breast cancer subtype^11,12^. The present analysis further characterizes the association between elevated inflammatory biomarkers and breast cancer relapse as being perhaps more relevant in subjects with HR+/HER2− tumors, and potentially less relevant in HER2+ tumors. This association in the HER2− group was attenuated by high-grade histology, suggesting an even stronger association in lower-grade, more hormonally driven tumors. Subjects for whom grade was not reported were included with patients with low-intermediate grade tumors, so that any potential misclassification would bias in favor of a null association.

The mechanism underlying the relationship between increased inflammation and increased risk of HR+/HER2− relapse on an AI remains unclear, though one proposed pathway is increased estrogen biosynthesis as a result of increased aromatase expression^8^. While we found that HR+/HER2− subjects with elevated CRP levels had numerically higher non-suppression rates and levels of serum estrogens, these values did not reach statistical significance. Therefore, if increased estrogen metabolism plays a role in the relationship between elevated serum inflammatory biomarkers and resistance to AIs, a better understanding of the underlying pathophysiology is needed, as it is possible this effect may be localized to breast tissue and not reflected in serum estrogen levels^16^. High-risk IL-6 promoter genotypes in the HR+/HER2− subgroup were associated with increased risk of relapse, as well as increased serum CRP and SAA, but these factors did not seem to influence estrogen metabolite levels.

While both elevated serum CRP and SAA were associated with an increased risk of breast cancer relapse, the biologic (additive) interaction analysis between these two biomarkers does not suggest a synergistic effect in which elevated levels of both biomarkers drastically increases one’s risk of relapse. The weakly negative RERI suggests a less than additive effect^17^, but not a necessarily antagonistic relationship. This finding is not surprising given what is known biologically about CRP and SAA. Both are produced primarily by the liver and are triggered by elevations in IL-6^10,18^. Of note, the inclusion of the cross-product CRP*SAA in the statistical (multiplicative) analysis merely facilitates an accurate inclusion of CRP and SAA individually in the multivariate model, and does not itself represent a “synergistic” effect. The interaction and multivariate analyses in this study demonstrate that the knowledge gained by measuring SAA in addition to CRP, if there is any, is minor. Therefore, serum CRP appears to be the most appropriate inflammatory biomarker to identify this subpopulation of high-risk patients.

In addition, CRP has already been used as part of an inflammatory risk score in the metastatic setting^19–21^. The modified Glasgow Prognostic Scale (mGPS) gives one point for elevated CRP >10.0 mg/L (similar to this study), and one point for serum albumin <35 g/L. Future studies seeking to identify subjects on the basis of an inflammatory mediated risk of relapsed breast cancer should therefore strongly consider checking CRP. While SAA may not provide additional predictive information, hypoalbuminemia may be a more global measure of inflammation, and the mGPS would be a reasonable scale to consider in helping identify this at-risk population.

While serum CRP, IL-6, and SAA levels were not associated with increased risk of breast cancer recurrence in the HER2+ group, there was a strong association of the presence of a high-risk IL-6 promoter genotype with increased risk of HR+/HER2+ breast cancer relapse. Pre-clinical evidence supports this finding. Korkaya et al.^22^ generated trastuzumab resistant HER2+ breast cancer cells by knocking down PTEN, and found that this resistance to trastuzumab was mediated by an IL-6 inflammatory feedback loop, resulting in increased levels of intracellular IL-6 and expansion of a cancer stem cell population. This expansion was reversed by introduction of an IL-6 receptor antibody interrupting the feedback loop. Therefore, the patients in this study with HER2+ tumors and a high-risk IL-6 promoter genotype may have been intrinsically resistant to adjuvant trastuzumab therapy due to increased transcription of intracellular IL-6. While serum inflammatory biomarkers may not be an effective means of identifying or monitoring such patients, these findings lend further support to targeting the IL-6/JAK/STAT pathway in some patients with HER2+ breast cancer who harbor the high-risk IL-6 genotype.

Though biomarkers are typically thought of as either being prognostic (in determining the metastatic potential and/or risk of relapse or progression regardless of treatment) or predictive (in determining the sensitivity of the tumor to a particular treatment), few biomarkers are truly one or the other^23^. Because all patients on this study were prescribed an AI for adjuvant therapy and received trastuzumab as part of their initial chemotherapy regimen if HER2+, high CRP and high-risk IL-6 promoter genotypes might be potential predictive biomarkers in their potential ability to predict response to AIs (in HR+/HER2− subjects) or trastuzumab (in HR+/HER2+subjects). However, a more detailed understanding of these underlying pathways and prospective evaluation is needed before such a classification could be implemented clinically. These data could be further validated in completed clinical trials if there are available blood samples for analysis. In the HR+/HER2− population, adjuvant trials using AIs with adequate follow up would be an ideal validation set. In the HER2+ population, assessment of patients’ IL6 promoter genotype could be performed in either adjuvant studies of HER2 targeted therapy with adequate survival follow up, or neoadjuvant studies with robust pathologic complete response data.

There are several strengths and limitations of this study. Because HR+ breast cancer relapse is a relatively uncommon event, a nested case-control design from a large prospective cohort is an appropriate, and feasible, design to measure the effects of exposure to pathways of resistance. Although “risk” is not technically measured in a case-control study, the OR approximates the risk ratio if the rare disease assumption is met in the population (which in this case it is, as relapsed patients represent about 3.5% of the overall cohort). Hence, the term “risk” can be used to describe the associations observed in this study. The use of different cutpoints for inflammatory biomarkers has been a criticism of some cancer epidemiologic studies, as this decreases generalizability^24^. When available, this study included clinically derived cutpoints based on clinical lab reference values, which improves the generalizability of the findings. An additional criticism of inflammatory biomarker studies is that the biomarkers can vary significantly throughout the day, depending on the time they are collected^24^. Though the collection time was not standardized in this study, samples were collected according to clinical convenience prior to relapse, and thus any bias is likely non-differential.

IL-6 levels, while previously shown to be associated with worse outcomes in patients with active breast cancer^25^, were not associated with an increased risk of relapse in the present analysis of HR+ breast cancer survivors. This potentially reflects the difference in half-lives between inflammatory cytokines like IL-6, which is measured on the order of hours^26^, as compared to their downstream acute phase proteins, like CRP and SAA, which can remain elevated for days and be detected more easily^18^.

Sensitivity analyses were performed to protect against some of the limitations of this study. While the long follow-up of this prospective cohort is a strength (median follow up of approximately 10 years from time of breast cancer diagnosis), patients enrolled later in the study had significantly less follow up time (as little as 3.5 years among non-relapsed subjects). The sensitivity analysis suggests that the directionality of the effect of the inflammatory biomarkers was not impacted by follow up time, though the magnitude of the effect was for serum SAA. Because the serum biomarker results were unchanged after excluding relapses that would have occurred in the first 6 years and after 18 years since diagnosis, it is unlikely that additional follow up time would change these findings. In addition, ignoring the matching criteria in the HER2− group did not significantly diminish the effect of the inflammatory biomarkers on the risk of relapsed breast cancer, suggesting that the multiple uses of individual controls did not significantly alter the results.

In conclusion, this study demonstrates that elevation in the serum inflammatory biomarkers CRP or SAA drawn randomly during treatment in the adjuvant setting are significantly and independently associated with increased risk of relapsed HR+/HER2− breast cancer, but not HR+/HER2+ breast cancer. This association is amplified in patients with low-intermediate grade tumors, suggesting a stronger association in tumors that are more likely to be strongly hormone receptor positive. The mechanism of inflammatory mediated AI resistance may be more localized at the tissue level, as serum estrogens were not impacted by increased inflammation. The presence of a high-risk IL-6 promoter genotype polymorphism is strongly and significantly associated with relapsed HR+/HER2+ breast cancer, and may have important therapeutic implications. Confirmatory studies are warranted that utilize these biomarkers to identify a subgroup of high-risk HR+/HER2− patients that may benefit from anti-inflammatory/anti-IL-6 therapy in the adjuvant setting. Patients with HER2+ breast cancer at risk for primary trastuzumab resistance could potentially be identified on the basis of a high-risk IL-6 promoter genotype, and future studies could be aimed at overcoming this resistance by inhibiting the IL-6 pathway.

## Methods

### Study designs

This was a nested case-control study, with cases and controls selected from a larger prospective cohort study (the Wellness After Breast Cancer-II Cohort, or WABC-II). WABC-II enrolled 1287 post-menopausal women treated within the University of Pennsylvania Health System for stage I–III, ER+ breast cancer, who had completed definitive surgery and adjuvant chemotherapy and/or radiotherapy as standard of care and were receiving adjuvant hormone therapy with an aromatase inhibitor. Subjects were enrolled from March 2008 through November 2013 and followed for recurrence; a comprehensive data lock was performed in August 2015. This study was approved by the Institutional Review Board of the University of Pennsylvania and all study procedures were conducted according to the institution’s code of ethics. Informed consent was obtained from all individual participants in the study.

Reporting of all results are in accordance with the REMARK guidelines^27^. Cases were defined as having recurred based on the standardized STEEP criteria for recurrence free interval (RFI)^28^, which includes invasive ipsilateral breast tumor recurrence, local/regional invasive recurrence, distant recurrence, or death from breast cancer. Subjects exited the cohort at the time of relapse. Controls were selected in a 1:5 case/control ratio using cumulative density sampling (also known as survivor sampling), and individually matched with replacement on three factors: time since diagnosis, time on aromatase inhibitor, and HER2 status. These matching criteria were chosen to minimize influence of the most important covariates, since increasing time since diagnosis, decreasing time on AI, and HER2+ status are all associated with an increased risk of relapsed breast cancer. Because 38 HER2− cases were available for analysis, a 1:5 case to control ratio was chosen to achieve 94% power to detect an Odds Ratio (OR) of 2.00, assuming an alpha of 0.05 and a prevalence of elevated biomarker levels in the controls of 30% (based on prior data^11^). In addition to recurrent breast cancer, clinical variables of interest included age, high-grade, >4 positive nodes, race, and obesity at diagnosis, defined as a body mass index (BMI) ≥30 kg/m^2^. High-grade was distinguished from low-moderate grade. If tumor grade was not specified in the pathology report, the grade was classified as low-moderate.

In March 2018, the relapse status of all controls was updated again to reduce the risk of misclassification, and controls for any new cases that were identified were assigned from the existing pool of controls based on the same matching criteria. The ER+/HER2+ subset of patients in the study represented a minority of the overall population (*n* = 74; 38%). These patients were therefore analyzed separately, and all ER+/HER2+ cases and controls were included in the analysis, rather than being individually matched.

Blood samples were drawn at the time of enrollment into WABC-II, processed to obtain serum, plasma and buffy coat, and subsequently stored at −80 °C until the time of analysis. Additional samples were taken at one year of routine clinical follow up if the patient was still enrolled in the cohort (i.e., had not yet relapsed). When available, the initial enrollment sample was used for the present analysis, with additional follow up samples used if the enrollment sample was missing. Subjects with both missing enrollment and follow up samples were excluded from the analysis, as these samples were missing at random.

### Inflammatory biomarker collection and measurement

The serum concentrations of CRP, IL-6, and SAA (primary exposure variables) were measured using the commercially available Luminex quantitative multiplex bead assay (R&D Systems, Minneapolis, MN) and was performed by the Human Immunology Core in the Perelman School of Medicine at the University of Pennsylvania. Because assay validation is key in proper biomarker analysis and reporting^27,29^, analysis of individual serum samples were performed in tandem pairs and back calculated against a standardized curve repeated on each plate analyzed. Laboratory staff performing the analyses were blinded to case/control status. Descriptive results of serum biomarker levels were reported both as median values and proportion of subjects with values greater than the upper limit of normal (>ULN). The ULN for the clinically utilized biomarkers CRP and IL-6 were defined from reference intervals that are based on the distribution in the general population and published online by the HUP lab test services guide, with an elevated CRP being defined as >7.5 mg/L^30^, and elevated IL-6 being defined as ≥5 pg/mL^31^. SAA is not currently utilized clinically, therefore no ULN was available for consideration with regard to this variable. Results were compared between cases and controls using the Wilcoxon rank-sum test (for median biomarker levels) and chi squared test (for proportion of subjects with values >ULN).

### Statistical analysis for inflammatory biomarkers

Because the HER2− cases and controls were individually matched, while all HER2+ cases and controls were selected, statistical analyses were performed separately for each cohort. Median serum concentrations of each inflammatory biomarker amongst cases and controls were compared using the Wilcoxon rank-sum test. Because the serum biomarker values were not normally distributed, a natural log transformation was performed to normalize the data (Fig. 1b) prior to any analyses that used these biomarkers as continuous variables.

Conditional logistic regression was utilized as the measure of association in the inflammatory biomarker analysis in the HER2− subgroup, and logistic regression was utilized in the HER2+ subgroup in the following steps. First, univariate logistic regression of each biomarker was performed separately for each of the candidate biomarkers. Second, for biomarkers showing a significant association in the univariate analysis, optimal cutpoints were created by performing a classification tree analysis. Classification tree analysis employs a standard algorithm that recursively divides the data into two groups on the basis of a dependent variable that maximizes the difference between the proportion of the event of interest between the two groups^32,33^. Receiver operating characteristic (ROC) curves were generated based on simple logistic regression to confirm the predictive ability of the biomarkers considered for classification tree analysis. Third, biomarkers found to be significantly associated with relapsed breast cancer were investigated for statistical interaction by creating a multivariate regression model that included the individual candidate biomarkers, as well as a cross-product term. To test for synergy on the additive scale, also known as biologic interaction^34–36^, the relative excess risk due to interaction (RERI) was calculated^17^. The RERI between two dichotomous biomarker variables was calculated by inserting variables that represent one of three scenarios into a (conditional) logistic regression model: two variables each representing an instance in which one biomarker is “positive” (above the cutpoint established in the second step above) while the other is negative, and one variable representing an instance in which both biomarkers are positive, with the common reference being subjects who are “negative” for both biomarkers. After this multivariable regression model was generated, beta coefficients and variance/covariance values were used to calculate the RERI^37^. Fourth, the adjusted association between relapsed breast cancer controlling for the clinical covariates of interest and the significant biomarkers were estimated using multivariate logistic regression. Lastly, a final multivariate regression model was built using the significant individual biomarkers, biomarker interactions, and covariates identified in the prior analyses. Final multivariate logistic regression models were manually constructed using forward selection, and confirmed using backward selection. Sensitivity analyses were performed to account for extremes in follow up time, as well as the use of individual controls multiple times. All descriptive and statistical tests, with the exception of the classification tree analysis, were performed using the Stata Statistical Software Package version 14.2 (StataCorp, College Station, TX). Classification tree analysis was performed using CART software (Salford Systems, San Diego, CA).

### IL-6 promoter genotyping and analysis

IL-6 promoter genotyping methods were adopted from previously described techniques^38^. The forward primer sequence was 5’ AAA AAG GAG TCA CAC ACT CCA CCT 3’ and the reverse primer sequence was 5’ TTG GGC TGA TTG GAA ACC TTA TTA 3’. The enzyme used for the PCR reactions was the Roche Expand High Fidelity PCR system (Cat. No 11 759 078 001) and the cycling conditions were as follows: 95C 5 min; 30 cycles of 95C 15 s, 57C 30 s, and 72C 30 s; 72C 5 min; then held at 4C. PCR products were purified with ExoSap. Sequencing reactions were then assembled using BigDye 3.1 (ThermoScientific) and sequenced on ABI 3730XL sequencer in both forward and reverse directions. DNA was extracted from buffy coat samples drawn at the time of enrollment into the WABC cohort and underwent Sanger sequencing for three functional variants of the IL-6 promoter: −572G>C (rs1800796), −597G>A (rs1800797), and −174G>C (rs1800795). Based on previously published data^9^, subjects were considered to have a high-risk IL-6 promoter genotype if a “G/G” genotype was present for the −597 and −174 variants. Of all 196 subjects, two samples were missing (one HR+/HER2− case and one HR+/HER2+ control). Conditional and simple logistic regression models were used respectively for the HER2− and HER2+ subgroups to measure the level of association of high-risk IL-6 genotypes with relapsed breast cancer. Confounding effects between clinical covariates and genotype profiles were assessed using logistic regression.

### Serum estrogen metabolite and aromatase inhibitor measurement and analysis

Estrone and estradiol standards were purchased from Steraloids Inc. (Newport, RI). [13C6]-estrone and [13C6]-estradiol were purchased from Cambridge Isotope Laboratories (Cambridge, MA). [13C3]-exemestane was purchased from Isosciences (Ambler, PA) and [2H3]-17β-hydroxy-exemestane, [2H4]-letrozole and [2H12]-anastrozole were purchased from Toronto Research Chemicals (Toronto, Ontario). β-glucuronidase/arylsulfatase (Helix pomatia) was obtained from Roche (Indianapolis, IN). Dry acetonitrile was purchased from Acros Organic (New Jersey, USA). Methyl-tert-butyl-ether (MTBE), 2-fluoro-1-methylpyridinium p-toluenesulfonate (FMP-TS), triethylamine, methanol, acetone, L-ascorbic acid, formic acid, hydrochloric acid (HCl), sodium chloride, sodium acetate and sodium bicarbonate were obtained from Sigma–Aldrich (Milwaukee, WI)^39^. Off the clot double charcoal-stripped human serum was purchased from Golden West Biologicals, Inc. (Temecula, CA). All solvents were HPLC Optima grade unless otherwise noted.

Off the clot double charcoal-stripped human serum was used as an analytical matrix for quantification of estrogen metabolites from human serum. An internal standard mix containing [13C6]-estrone, [13C6]-estradiol, [13C3]-exemestane, [2H3]-17β-hydroxy-exemestane, [2H4]-letrozole and [2H12]-anastrozole was spiked into serum prior to extraction. Calibration curves of estrogens were prepared from standard solutions in the range of 1.56–800 pg/mL. For determination of total estrogens, 10 µL of internal standard working solution was spiked into a 0.1 mL aliquot of serum, followed by the addition of 0.1 mL water, 0.1 mL 0.5% L-ascorbic acid, 0.2 mL sodium acetate buffer (200 mM, pH 5.0), and 20 µL of β-glucuronidase/arylsulfatase. Samples were incubated at 37 °C for 19 h. After hydrolysis, samples were acidified with 15 µL of 1 N HCl followed by addition of 150 µL saturated sodium chloride. Samples underwent liquid–liquid extraction (LLE) with 2.5 mL of MTBE by vortex-mixing for 20 min, followed by centrifugation at 3400 × *g* at 4 °C for 15 min. The upper, organic layer containing extracted estrogens was removed and dried under nitrogen prior to chemical derivatization and LC-HRMS analysis. Formation of methylpyridinium ether derivatives of estrone and estradiol proceeded as follows. 2-fluoro-1-methylpyridinium p-toluenesulfonate (FMP-TS) reagent was freshly prepared at 5 mg/mL in acetonitrile containing 1% triethylamine. Fifty microliter was added to each vial containing extracted estrogens. The mixture was vortexed for 10 s and then incubated at 45 °C for 15 min. The reaction was stopped by the addition of 50 µL water containing 0.1% formic acid and 5 µL of this mixture was directly injected for LC-HRMS analysis.

Separations were performed on a Waters BEH C18 Column (2.1 mm × 50 mm 1.7 μm) using a 7 min gradient starting at 65% methanol w/ 0.1% formic acid. Mobile phase A was water with 0.1% formic acid, and mobile phase B was methanol with 0.1% formic acid. A Thermo QExactive HF instrument was operated in positive ion mode alternating full scan and MS/MS modes at 120,000 resolution. The MS was coupled to an Ultimate 3000 UHPLC interfaced with a heated electrospray ionization (HESI-II) source. Molecular (M+) precursor ions of estrogens were as follows: estrone: 362.2115; [13C6]-estrone: 368.2361; estradiol: 364.2271; and [13C6]-estradiol: 370.2465. The method used the separation of signal from noise based on the molecular ion’s unique stability, by applying extra CID on the parent ion. Aromatase inhibitors and corresponding internal standard molecular ions (M+) were detected in full scan according to accurate mass as follows: letrozole: 286.1087; [2H4]-letrozole: 290.1333; anastrozole: 294.1713; [2H12]-anastrozole: 306.2459; exemestane: 297.1847; [13C3]-exemestane: 300.1948; 17β-hydroxy-exemestane: 299.1996; [2H3]-17β-hydroxy-exemestane: 302.2184. Serum concentrations of estrogens were calculated using Xcalibur software (version 3.0) from Thermo Fisher Scientific. The limit of detection was 1.0 pg/mL for both E1 and E2. Laboratory staff performing the analyses were blinded to case/control status.

Serum estrogens were reported in both qualitative (estrogens detected or not) and quantitative fashions. Subjects with serum E1 or E2 levels that were below the limit of detection were reported as 0.2 pg/mL (or the limit of detection/5) so they could be included in the quantitative analysis. Serum estrogen levels and high inflammatory states were compared by measuring the proportion of detectable estrogen metabolites, as well as mean estrogen level within each group. Statistical significance for any differences observed was measured using chi squared (detectable estrogens) and Wilcoxon rank-sum (mean estrogen levels) tests.

A total of 12 subjects had hormone serum samples missing at random (6 HER2+ controls, 2 HER2+ cases, 3 HER2− controls, and 1 HER2− case) and were therefore not included in the analysis. 14 subjects had their serum estrogens measured as part of a pilot project in 2010 to measure serum estrogen levels. Those results are included as part of the qualitative analysis only given a subsequent refinement of laboratory techniques that more precisely quantifies the serum estrogen levels, and were only included if no additional serum samples remained for repeat testing.

### Reporting summary

Further information on experimental design is available in the Nature Research Reporting Summary linked to this paper.

## Supplementary information

Supplementary Table 1

Reporting Summary Checklist FLAT

## Data Availability

The data generated and analyzed during this study are openly available in both Stata and open (.xlsx) formats as part of the following data record: 10.6084/m9.figshare.12954533^40^.
