# Supplementary material for: Effects of systemic inflammation on relapse in early breast cancer
Source: NPJ Breast Cancer. 2021 Jan 22;7:7. doi: 10.1038/s41523-020-00212-6 (PMC7822844; doi:10.1038/s41523-020-00212-6)
Supplement: Supplementary file 1 — Supplementary Table 1 [file 41523_2020_212_MOESM1_ESM.pdf]

Supplementary Table 1: Estrogen analyses overall and by CRP and IL6 promoter status in HER2+ subjects

	**Estrogens Detectable (%)**			**Mean Estrogen Values (pg/mL)**		
**Estrogen**	**Controls**	**Cases**	**p**	**Controls**	**Cases**	**p**
E1	29%	63%	0.028	28.5	57.4	0.126
E2	15%	44%	0.019	1.4	3.8	0.212
**Estrogen**	**CRP ≤ median**	**CRP > median**	**p**	**IL6 Promoter +**	**IL6 Promoter -**	**p**
E1 Detectable (%)	32%	38%	0.66	41%	28%	0.27
E2 Detectable (%)	18%	22%	0.66	29%	9%	0.04
E1 (pg/mL), mean	22.3	41.1	0.94	26.8	37.5	0.61
E2 (pg/mL), mean	1.4	1.9	0.79	2.4	1.1	0.20
