# Supplementary material for: Effects of systemic inflammation on relapse in early breast cancer
Source: NPJ Breast Cancer. 2021 Jan 22;7:7. doi: 10.1038/s41523-020-00212-6 (PMC7822844; doi:10.1038/s41523-020-00212-6)
Supplement: Supplementary file 2 — Reporting Summary Checklist FLAT [file 41523_2020_212_MOESM2_ESM.pdf]

nature research

Corresponding author(s): Angela DeMichele (submitting/first author Nicholas McAndrew)

Last updated by author(s): Jul 9, 2020

Reporting Summary

Nature Research wishes to improve the reproducibility of the work that we publish. This form provides structure for consistency and transparency in reporting. For further information on Nature Research policies, see our Editorial Policies and the Editorial Policy Checklist.

Statistics

For all statistical analyses, confirm that the following items are present in the figure legend, table legend, main text, or Methods section.

n/a	Confirmed	
☐	☒	The exact sample size (n) for each experimental group/condition, given as a discrete number and unit of measurement
☐	☒	A statement on whether measurements were taken from distinct samples or whether the same sample was measured repeatedly
☐	☒	The statistical test(s) used AND whether they are one- or two-sided<br>*Only common tests should be described solely by name; describe more complex techniques in the Methods section.*
☐	☒	A description of all covariates tested
☐	☒	A description of any assumptions or corrections, such as tests of normality and adjustment for multiple comparisons
☐	☒	A full description of the statistical parameters including central tendency (e.g. means) or other basic estimates (e.g. regression coefficient) AND variation (e.g. standard deviation) or associated estimates of uncertainty (e.g. confidence intervals)
☐	☒	For null hypothesis testing, the test statistic (e.g. F, t, r) with confidence intervals, effect sizes, degrees of freedom and P value noted<br>*Give P values as exact values whenever suitable.*
☒	☐	For Bayesian analysis, information on the choice of priors and Markov chain Monte Carlo settings
☒	☐	For hierarchical and complex designs, identification of the appropriate level for tests and full reporting of outcomes
☒	☐	Estimates of effect sizes (e.g. Cohen's d, Pearson's r), indicating how they were calculated

Our web collection on statistics for biologists contains articles on many of the points above.

Software and code

Policy information about availability of computer code

Data collection	No softward was used.
Data analysis	All descriptive and statistical tests, with the exception of the classification tree analysis, were performed using the Stata Statistical Software Package version 14.2 (StataCorp, College Station, TX). Classification tree analysis was performed using CART software (Salford Systems, San Diego, CA).

For manuscripts utilizing custom algorithms or software that are central to the research but not yet described in published literature, software must be made available to editors and reviewers. We strongly encourage code deposition in a community repository (e.g. GitHub). See the Nature Research guidelines for submitting code & software for further information.

Data

Policy information about availability of data

All manuscripts must include a data availability statement. This statement should provide the following information, where applicable:

- Accession codes, unique identifiers, or web links for publicly available datasets
- A list of figures that have associated raw data
- A description of any restrictions on data availability

The dataset analyzed in the current study is available from the corresponding author upon reasonable request.

Field-specific reporting

Please select the one below that is the best fit for your research. If you are not sure, read the appropriate sections before making your selection.

☒ Life sciences ☐ Behavioural & social sciences ☐ Ecological, evolutionary & environmental sciences

For a reference copy of the document with all sections, see nature.com/documents/nr-reporting-summary-flat.pdf

Life sciences study design

All studies must disclose on these points even when the disclosure is negative.

Sample size	Because 38 HER2- cases were available for analysis, a 1:5 case to control ratio was chosen to achieve 94% power to detect an Odds Ratio (OR) of 2.00, assuming an alpha of 0.05 and a prevalence of elevated biomarker levels in the controls of 30% (based on prior data).
Data exclusions	Subjects with both missing enrollment and follow up samples were excluded from the analysis, as these samples were missing at random.
Replication	Replication of biomarker measurements was not possible because the majority of patients only had one sample available for testing.
Randomization	Not applicable.
Blinding	Not applicable.

Reporting for specific materials, systems and methods

We require information from authors about some types of materials, experimental systems and methods used in many studies. Here, indicate whether each material, system or method listed is relevant to your study. If you are not sure if a list item applies to your research, read the appropriate section before selecting a response.

Materials & experimental systems

n/a	Involved in the study	
☒	☐	Antibodies
☒	☐	Eukaryotic cell lines
☒	☐	Palaeontology and archaeology
☒	☐	Animals and other organisms
☐	☒	Human research participants
☐	☒	Clinical data
☒	☐	Dual use research of concern

Methods

n/a	Involved in the study	
☒	☐	ChIP-seq
☒	☐	Flow cytometry
☒	☐	MRI-based neuroimaging

Human research participants

Policy information about studies involving human research participants

Population characteristics	WABC-II enrolled 1287 post-menopausal women treated within the University of Pennsylvania Health System for stage I – III, ER+ breast cancer, who had completed definitive surgery and adjuvant chemotherapy and/or radiotherapy as standard of care and were receiving adjuvant hormone therapy with an aromatase inhibitor. Subjects were enrolled from March 2008 through November 2013 and followed for recurrence; a comprehensive data lock was performed in August 2015.
Recruitment	Participants were recruited directly by their treating physicians.
Ethics oversight	This study was approved by the Institutional Review Board of the University of Pennsylvania and all study procedures were conducted according to the institution's code of ethics. Informed consent was obtained from all individual participants in the study.

Note that full information on the approval of the study protocol must also be provided in the manuscript.

Clinical data

Policy information about clinical studies

All manuscripts should comply with the ICMJE guidelines for publication of clinical research and a completed CONSORT checklist must be included with all submissions.

Clinical trial registration	This was not a clinical trial, but a prospective cohort observational study.
Study protocol	The full study protocol for the WABC-II cohort can be made available to the editorial board upon request.

Data collection	All data was collected at the University of Pennsylvania.
Outcomes	Cases were defined as having recurred based on the standardized STEEP criteria for recurrence free interval (RFI)28, which includes invasive ipsilateral breast tumor recurrence, local/regional invasive recurrence, distant recurrence, or death from breast cancer.
